# Practical value of three-dimensional high resolution magnetic resonance Vessel Wall imaging in identifying suspicious intracranial vertebrobasilar dissecting aneurysms

**DOI:** 10.1186/s12883-020-01779-0

**Published:** 2020-05-20

**Authors:** Xianjin Zhu, Hancheng Qiu, Ferdinand K. Hui, Yiqun Zhang, Yun-e Liu, Fengyuan Man, Wei-Jian Jiang

**Affiliations:** 1 Department of Neurology, Beijing Friendship Hospital, Capital Medical University, No. 95 Yong An Road, Xicheng District, Beijing, 100050 China; 2 New Era Stroke Care and Research Institute, The PLA Rocket Force Characteristic Medical Center, No. 16 Xinjiekouwai Street, Xicheng District, Beijing, 100088 China; 3 Department of Radiology, Johns Hopkins Hospital, Baltimore, MD USA; 4 Department of Radiology, The PLA Rocket Force Characteristic Medical Center, Beijing, China

**Keywords:** Dissecting aneurysm, Vertebrobasilar artery, High resolution magnetic resonance image, Vessel wall imaging

## Abstract

**Background:**

Direct evidence of intimal flaps, double lumen and intramural haematomas (IMH) is difficult to detect on conventional angiography in most intracranial vertebrobasilar dissecting aneurysms (VBDAs). Our purpose was to assess the value of three-dimensional high-resolution magnetic resonance vessel wall imaging (3D HRMR VWI) for identifying VBDAs.

**Methods:**

Between August 2013 and January 2016, consecutive patients with suspicious VBDAs were prospectively enrolled to undergo catheter angiography and VWI (pre- and post-contrast). The lesion was diagnosed as definite VBDA when presenting direct signs of dissection; as possible when only presenting indirect signs; and as segmental ectasia when there was local dilation and wall thickness similar to adjacent normal artery’s without mural thrombosis.

**Results:**

Twenty-one patients with 27 lesions suspicious for VBDAs were finally included. Based on findings of VWI and catheter angiography, definite VBDA was diagnosed in 25 and 7 lesions (92.6%, vs 25.9%, *p* <  0.001), respectively; possible VBDA in 0 and 20 (0 vs 74.1%), respectively; and segmental ectasia in 2 and 0 (7.4% vs 0%), respectively. On VWI and catheter angiography, intimal flap was detected in 21 and 7 lesions (77.8% vs 25.9%, *p* = 0.001), respectively; double lumen sign in 18 and 7 (66.7% vs 25.9%, *p* = 0.003), respectively; and IMH sign in 14 and 0 (51.9% vs 0), respectively.

**Conclusions:**

3D HRMR VWI could detect direct dissection signs more frequently than catheter angiography. This may help obtain definite diagnosis of intracranial VBDAs, and allow accurate differentiation between dissecting aneurysm and segmental ectasia as well. Further prospective study with larger sample was required to investigate the superiority of HRMR VWI for definite diagnosis of intracranial VBDAs than catheter angiography.

## Background

Intracranial vertebrobasilar dissecting aneurysms (VBDAs) are not uncommon, and may exhibit intimal flaps, double lumens and intramural haematoma (IMH) [1]. Catheter angiography could provide dynamic images with good spatial resolution, and has long been regarded as the gold standard for the diagnosis of arterial dissection. However, catheter angiography generally provides intensity projection images, not the cross-sectional images, which limits the capability for detecting these characteristic signs of dissection [2, 3], and often reveals pearl and string sign, fusiform or serpentine dilation, even perhaps an arterial occlusion [3].

High resolution magnetic resonance vessel wall imaging (HRMR VWI) could provide cross-sectional images, and has been increasingly used in intracranial artery diseases [4]. Using two dimensional fast spin echo (2D FSE) sequences and black blood technique, the intracranial arterial wall can be visualized with greater sensitivity for the detection of vessel features suggestive or diagnostic of dissection [2, 5]. However, the identification may be impaired given the inherent curvature of intracranial arteries, limited anatomic coverage, and anisotropic spatial resolution [5, 6], leading to false-negative results, especially for cases involving 2 or more vessels [3].

An efficient 3D VWI technique, called Sampling Perfection with Application optimized contrasts by using different flip angle Evolutions (SPACE), was introduced in recent years [7], providing volumetric datasets that can be retrospectively reformatted for viewing in arbitrary orientations, and used for imaging multiple intracranial lesions simultaneously. Our purpose was to assess the practical value of 3D HRMR VWI with SPACE sequence in resolving vessel segments suspicious for VBDAs.

## Methods

### Patient selection

All the patients were consecutively enrolled from August 2013 to January 2016 to undergo catheter angiography and 3D HRMR VWI (pre- and post-contrast), when they fulfilled the following criteria: 1) clinical symptoms and findings on computed tomographic angiography (CTA) or MRA suggestive of an intracranial VBDA; 2) no contraindication for MR imaging and catheter angiography. Demographics, clinical findings, imaging data, and risk factors such as hypertension, hyperlipidemia, diabetes mellitus, and cigarette smoking were also collected prospectively. Atherosclerotic intraplaque hemorrhage (IPH), atherosclerotic ulceration and aneurysm with wall atherosclerosis that might mimic dissection were excluded from this study. The stages were classified as early stage (less than 2 weeks, and 2 weeks to 2 months) and chronic stage (longer than 2 months) based on the time between symptom onset to VWI examination.

### Catheter angiography protocol

All patients underwent catheter angiography on an Allura Xper FD20/10 (Philips Healthcare), Artis Zee Biplane, or Artis Zeego angiography unit (Siemens Healthcare). Catheter angiography on anteroposterior and lateral projections, and oblique projections as necessary were performed after placement of a diagnostic catheter in the V1 segment of the vertebral artery in question with injection of 5 ml of contrast (Iodixanol, GE healthcare) at a rate of 3 ml per second at 300 Pa pressure. Then, 3D angiography was performed while injecting a total 18 ml contrast at a rate of 3 ml per second at 300 Pa pressure.

### Catheter angiography assessment

Direct and indirect signs of VBDAs were assessed by two experienced interventional neuroradiologists on both conventional and 3D catheter angiography images independently, blinded to the clinical and other imaging information including CTA, MRA and VWI. Analytical data was used to calculate intraobserver and interobserver’s agreement. The differences between two observers were solved by consensus. Direct signs included visualization of an intimal flap, or a double lumen. Indirect signs included long filiform or irregular stenosis, occlusion; or fusiform or serpentine aneurysmal dilation with focal, long filiform, or irregular stenosis (pearl-and-string sign); or fusiform or aneurysmal dilation at a non-branching site [2].

### MRI protocol

MR exam was performed with a 3 T MRI scanner (Siemens, Magnetom Skyra, Erlangen, Germany) using a 20-channel phased-array head and neck joint coil. A 3D time-of-flight (TOF) MRA was first acquired to localize the intracranial arteries with the following parameters: TR/TE = 20/3.4 ms, FOV = 192 × 240 mm, thickness = 1 mm, matrix = 460 × 640, and NEX = 1, the voxel volume was 0.4 × 0.4 × 1 mm on MRA. HRMR VWI was acquired with a 3D SPACE T1W sequence in the coronal plane (45-mm-thick slab) to cover the major intracranial vessels as identified on the TOF MRA with the following parameters: TR/TE = 600/20 ms, FOV = 182 × 220 × 45 mm, matrix = 212 × 256 × 50, average = 1.5, echo train duration = 118, the voxel volume was 0.9 × 0.9 × 0.9 mm on SPACE T1W image. SPACE post-contrast T1WI was obtained 5 min after gadolinium injection (0.1 mmol/kg of gadopentetate dimeglumine, Magnevist; Bayer Schering Pharma, Berlin, Germany) with the same parameters as pre-contrast T1WI.

### MRI reformation

**Fig. 1:**
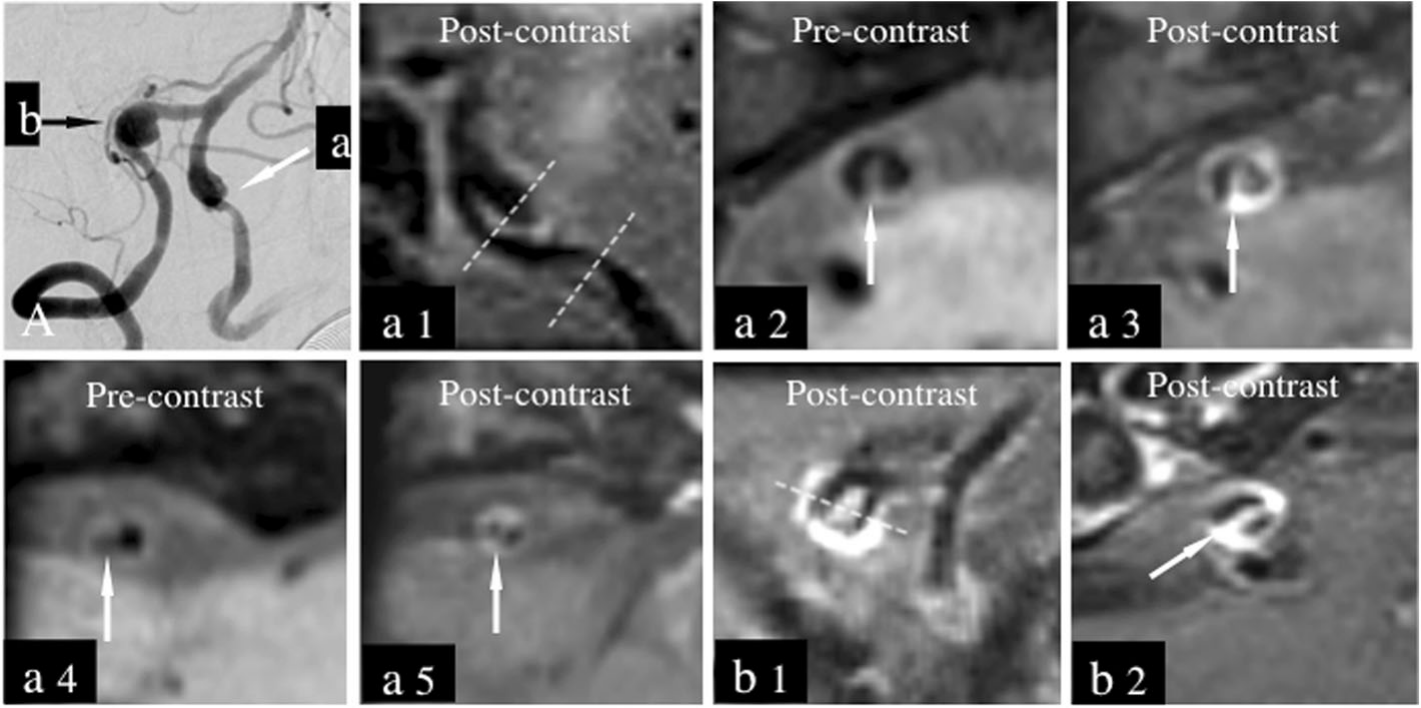
Patient 2. A 51-year-old man complained of dizziness and extremities weakness. Initial MRI was performed 11 days after symptom onset. Catheter angiography showed a lesion with a pear-and-string appearance (**a**, white arrow [**a**]) in the left intracranial vertebral artery and a lesion with aneurysmal dilation appearance (**a**, black arrow [**b**]) in the right side. VWI can be used for detecting the dissecting signs of both lesions simultaneously (a1-a5 for left side, b1-b2 for right side). Long-axis view of the lesion of left side revealed intimal flap and double lumen signs (a1, spotty line) on both the dilation and stenosis segments. Pre-contrast (a2, arrow) and post-contrast (a3, arrow) VWI showed intimal flap conspicuously on the dilation segment of left side (reconstructed at spotty line showed in a1). On the stenosis segment, intimal flap and double lumen was not detected on pre-contrast image (only eccentric wall thickness, a4, arrow) but on post-contrast image (a5, arrow). Long-axis view (b1, spotty line) and short-axis view (b2, arrow) of the lesion of right side revealed intimal flap and double lumen signs obviously

Resource data of VWIs were reformatted to acquire long and short axial images of the vertebrobasilar artery, with voxel volume = 0.9 × 0.9 × 0.9 mm. Multiple lesions with varying locations and orientations were displayed with individually reconstructed projections (Fig. 1a1-a5, b1-b2).

### MRI assessment

**Fig. 2:**
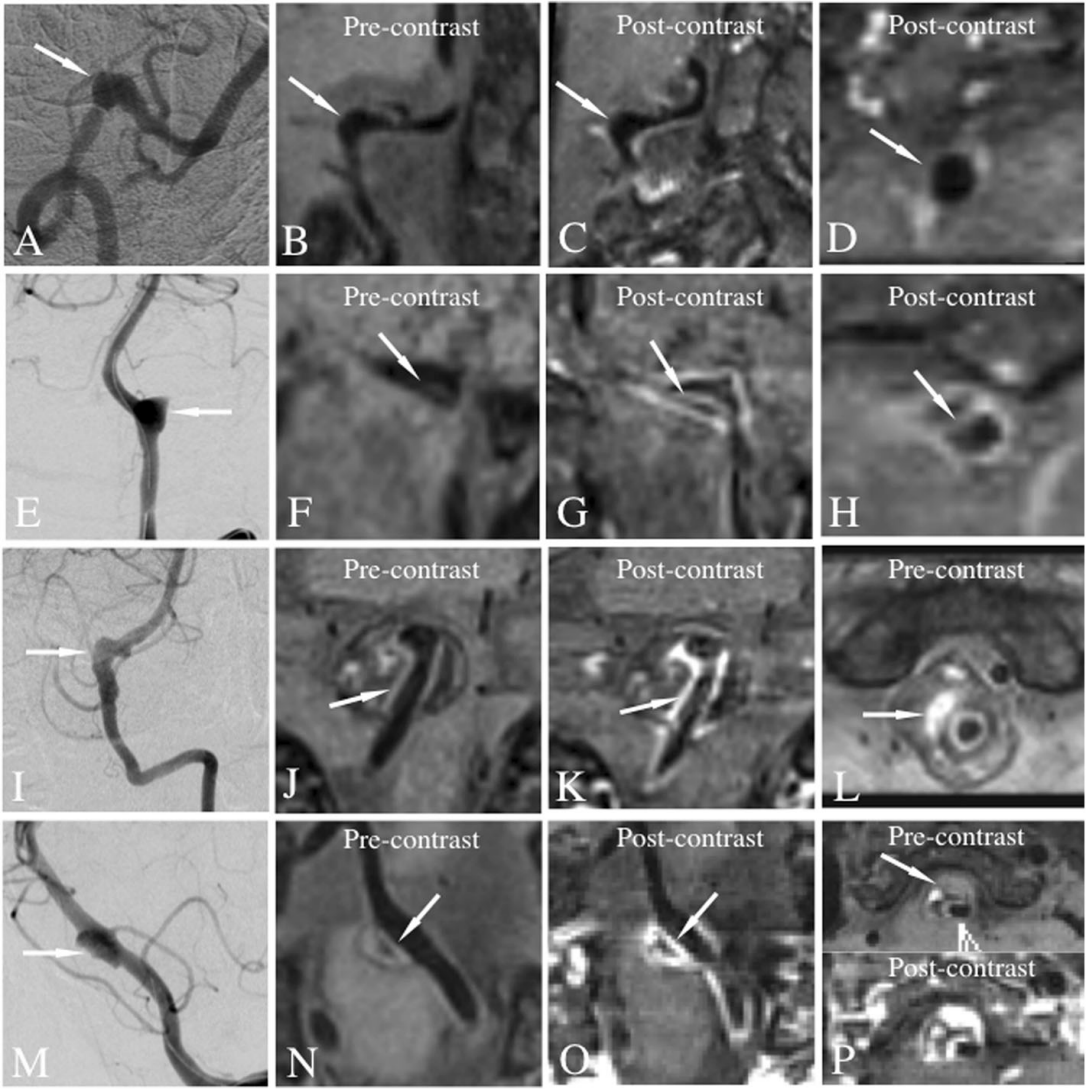
Similar angiographic finding (fusiform or aneurysmal dilation) had different pathognomonic findings on VWI. **a-d**, Patient 21. A 59-year-old man had suffered right side weakness and dysphagia for 20 days with final diagnosis of segmental ectasia. Catheter angiography showed fusiform dilation of the lumen without direct dissection signs (**a**, arrow). VWI also showed luminal dilation without intraluminal or extraluminal thrombus formation on pre-contrast (**b**, arrow) and post-contrast (**c**, **d**, arrow) images. **e-h**, Patient 13. A 40-year-old man had unconsciousness for 4 h with final diagnosis of dissection. Catheter angiography showed local dilation of the lumen (E, arrow) without direct dissection signs. VWI showed intimal flap and double lumen signs obscurely on pre-contrast (**f**, arrow) image, but obviously on post-contrast images (**g**, **h**, arrow). **i-l**, Patient 16. A 49-year-old man had complained recurrent dizziness for 2 years, with the symptom recurrence for 1 day. Catheter angiography also showed fusiform dilation of the lumen without direct dissection signs (**i**, arrow) as above cases. VWI showed mixed haematoma and intimal flap (**j**-**k**, arrow) between haematoma and signal void of flow. On the short-axis view of the haematoma demonstrated subacute blood in the chronic haematoma (L, arrow), strongly suggesting chronic dissection. **m-p,** Patient 6. A 66-year-old female have complained recurrent dizziness for 4 years. Catheter angiography showed a lesion with a dilation-without-stenosis appearance on the left vertebral artery (M, arrow). Short-axis view of pre-contrast (N, arrow) and post-contrast (O, arrow) VWI showed dissection with intimal flap and double lumen. Short-axis view of the lesion showed chronic and subacute haematoma (P, arrow). Post-contrast VWI showed dense and thick enhancement of the intimal flap and chronic haematoma (P, bottom row)

**Fig. 3:**
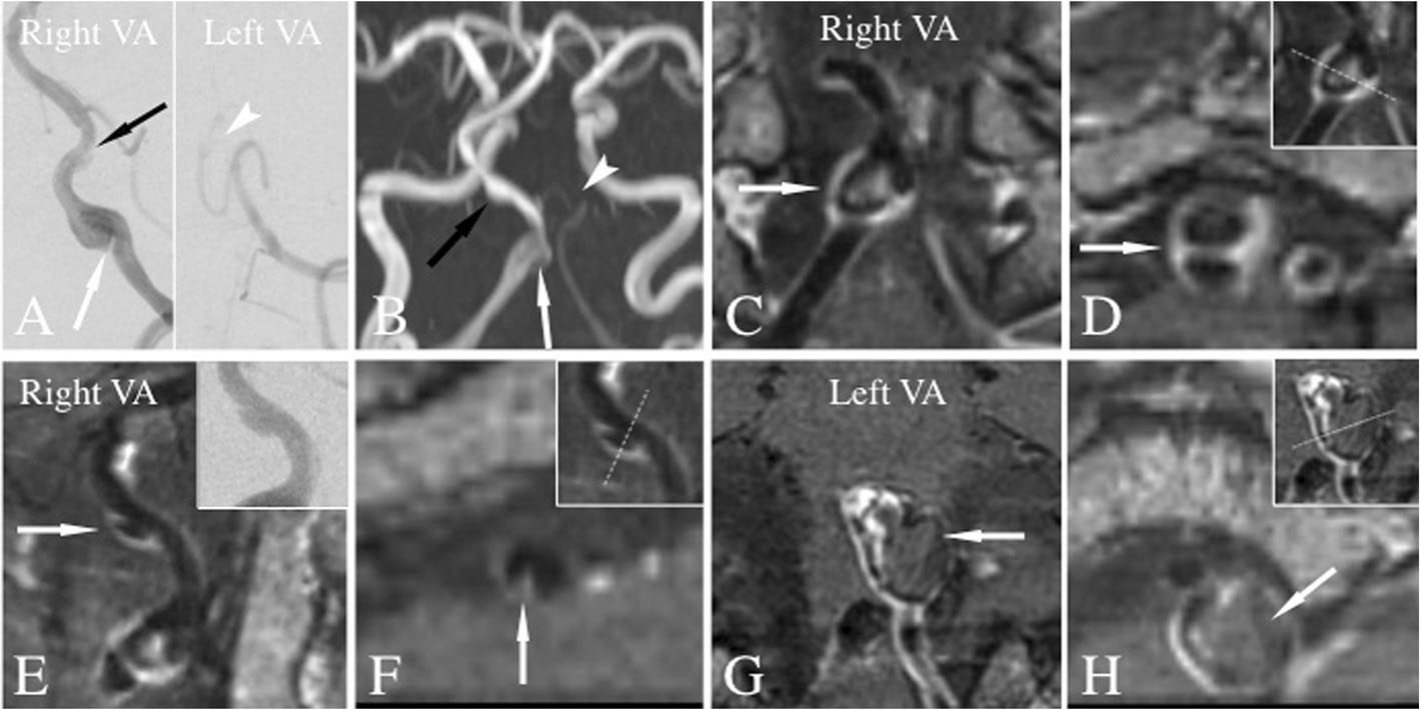
Patient 19. Multiple VBDAs affecting bilateral vertebral arteries (VAs). A 49-year-old man complained of recurrent headache for more than 1 year. Catheter angiography of right VA showed intimal flaps (**a**, white and black arrow) in 2 lesions with angiographic finding of aneurysmal dilation. Left distal VA seemed to be absent on catheter angiography suggesting hypoplasia or acquired occlusion (**a**, white arrow head). TOF-MRA showed similar luminal finding as catheter angiography. For the proximal lesion of right VA, long- (**c**, arrow) and short-axis (**d**, arrow) reconstructed view of post-contrast VWI demonstrated intimal flap and double obviously. Similar findings were detected for the distal lesion of right VA (**e**, **f**, arrow). For the left VA, long- (**g**, arrow) and short-axis reconstructed view (H, arrow) of post-contrast VWI revealed obvious dilation of outer contour and chronic haemotoma corresponding to the distal portion of left VA, suggesting acquired occlusion duo to dissection

Direct and indirect signs of VBDAs were assessed by 2 neuroradiologists independently with at least 5 years of experience in reading intracranial HRMR VWI, and were blinded to the clinical and other imaging information including CTA, MRA and catheter angiography images. Analytical data was also used to calculate intraobserver and interobserver’s agreement. The differences between two observers were solved by consensus. The direct signs included intimal flap, double lumen and IMH. Intimal flap sign was defined as an abnormal linear structure separating a true lumen from a false lumen (Figs. 1, 2g, k, o, 3d). To discriminate the intimal flap from the inflow artifact that can appear at the center of the lumen, we deemed only linear structures as an intimal flap when it extended to and from the arterial sidewall on SPACE pre- or post-contrast T1W images [2]. Double lumen sign was defined as two lumens represented as two jets of flow void within one vessel on MRI. This is distinct from fenestration, which are separate vessels. IMH sign was defined as detection of eccentric or circumferential hyper-, iso-, or hypointense signal (corresponding to hemorrhage age) on pre-contrast T1WI within the arterial wall (Fig. 2l, p) [ 8], generally extending longitudinally and located in the proximal or distal segment of pseudolumen, or filled the pseudolumen entirely. Intramural, partially thrombosed haematoma (Mizutani type 3 dissection) was diagnosed on imaging when heterogenous, multi-aged hemorrhage in the vessel wall was identified [9, 10], which is classified into IMH.

IMH was differentiated from intraluminal thrombus by location and enhancement: IMH is separated from the lumen by the intimal flap with no or sometimes heterogeneous enhancement. Lesions were considered intraluminal thrombus when it was seen in a juxtaluminal location with obvious enhancement [11]. Atherosclerotic IPH and ulceration which may mimic dissection was discriminated from IMH and excluded from this study with the following criteria: IPH and ulceration was still located within the atherosclerotic plaque with relatively small size, generally not associated with a focal enlargement of external diameter [3]. Atherosclerosis within an aneurysmal wall which may mimic chronic IMH was determined and excluded with the following characteristics including: at sites of arterial bifurcation, not associated with intimal flap and double lumen, not associated with heterogeneous T1 hyperintense signal (recurrent hemorrhage).

The indirect signs were also assessed based on the luminal characteristics of VWI, similar to that of catheter angiography.

### Lesion diagnosis

After assessment on catheter angiography and VWI, segments suspicious for VBDAs were given a final imaging diagnosis, individually. Based on catheter angiography, lesion was diagnosed as definite VBDAs by interventional neuroradiologists when intimal flap or double lumen was detected. On VWI, when IMH, intimal flap, or double lumen was found, it was considered a definite VBDA by neuroradiologists. If there were only indirect signs of dissection, the lesion was considered a possible VDBA. A lesion was considered segmental ectasia (Mizutani type 2), if there was only local dilation and normal wall thickness, without evidence of mural thrombosis [10].

### Statistical analysis

Continuous variables were described as mean ± standard deviation, or as interquartile range (if not normally distributed). Cohen’s k-statistic was computed to quantify the intra-observer and inter-oberver agreement for asessement of itimal flap, double lumen and IMH. A value of k > 0.75 was used to indicate a high level of agreement. After consensus, McNemar test was used to assess the difference for definitively diagnosing VBDAs and detecting direct dissection signs between VWI and catheter angiography. SPSS 11.5 (SPSS, Inc., Chicago, IL) was used for statistical analysis. All reported *p* values were 2-sided, and a p value of <0.05 was considered significant.

## Results

### Patients

Twenty-one consecutive patients (Male: 14; female: 7) with 27 vessel segments suspicious for intracranial VBDAs were included in this single center study. The median age was 59.0 years (interquartile range: 49.0 to 62.5 years). Risk factors included hypertension (*n* = 13, 61.9%), hyperlipidemia (*n* = 5, 23.8%), diabetes mellitus (*n* = 2, 9.5%), and cigarette smoking (*n* = 9, 42.9%). Of the 27 lesions, 8 lesions were located in the basilar artery, 16 lesions in the intracranial vertebral artery, and 3 lesions arising from the intracranial vertebral artery and extending into the basilar artery. Among the 21 patients, 3 patients presented with acute infarction in the territory of the parent artery, 17 with TIA, and 1 with subarachnoid hemorrhage (SAH). The mean interval between symptom onset and VWI examination was 30 days (interquartile range: 23 to 75.5 days). The patients were classified into early stage in 14 patients with 17 lesions (less than 2 weeks in 4 patients with 5 lesions, 2 weeks to 2 months in 10 patients with 12 lesions), and chronic stage (longer than 2 months) in 7 patients with 10 lesions. Catheter angiography was performed before VWI in 4 patients, after VWI in 16 patients, and on the same day with VWI in 1 patient. The median time between catheter angiography and MRI performance was 5 days (interquartile range: 1.5 to 7.5 days).

### Intraobserver and interobserver agreement

On catheter angiography, the intraobserver agreement was high for detecting an intimal flap (*k* = 0.899) and double lumen (*k* = 0.899). The interobserver agreement was aslso high for detecting an intimal flap (*k* = 0.807) and double lumen (*k* = 0.807).

On VWI, the intraobserver agreement was high for detecting intimal flap (*k* = 0.899), double lumen (*k* = 0.833) and IMH (*k* = 0.852). The interobserver agreement was also high for detecting intimal flap (*k* = 0.886), double lumen (*k* = 0.824) and IMH (*k* = 0.777).

### Final diagnosis

Catheter angiography and VWI data were available in all of the 27 vascular segments in question. Based on catheter angiography, 7 lesions in 7 patients were diagnosed as definite VBDAs with direct visualization of both an intimal flap and double lumen sign. There were 20 lesions in 14 patients that were deemed possible VBDAs with only indirect signs of dissection (Tables 1, 2).

**Table 1:** Comparison of catheter angiography and VWI for detecting dissecting signs and diagnosis

	Catheter angiography	VWI	*P*
Dissecting signs			
Intimal flap, n (%)	7 (25.9%)	21 (77.8%)	0.001
Double lumen, n (%)	7 (25.9%)	18 (66.7%)	0.003
IMH, n (%)	–	14 (51.9%)	
Diagnosis			
Definitive VBDA, n (%)	7 (25.9%)	25 (92.6%)	< 0.001
Possible VBDA, n (%)	20 (74.1%)	0	
Segmental ectasia, n (%)	0	2	

*VWI* vessel wall images, *IMH* intramural haematoma, *VBDA* vertebrobasilar dissecting aneurysm

**Table 2:** Dissecting signs on catheter angiography, and VWI for the individual patient and lesion

Patient No.	Symptoms and time from onset to imaging	Lesion location	Catheter angiography			VWI		
			Direct sign	Indirect sign	Diagnosis	Direct sign	Indirect sign	Diagnosis
1	Diplopia, 90 days	BA	–	Fusiform dilation	Possible VBDA	IMH	Fusiform dilation	VBDA
2	Dizziness and limb weakness, 10 days	Left ICVA	–	Fusiform dilation with stenosis	Possible VBDA	Intimal flap/ double lumen/ IMH	Fusiform dilation with stenosis	VBDA
		Right ICVA	–	Aneurysmal dilation	Possible VBDA	Intimal flap/ double lumen	Aneurysmal dilation	VBDA
3	Recurrent vertigo, 61 days	Left ICVA to BA	Intimal flap/double lumen	Fusiform dilation with stenosis	VBDA	Intimal flap/ double lumen	Fusiform dilation with stenosis	VBDA
4	Limb numbness and weakness, 30 days	Right ICVA to BA	–	Fusiform dilation with stenosis	Possible VBDA	Intimal flap/ IMH	Fusiform dilation with stenosis	VBDA
5	Slurred speech, 26 days	Left ICVA to BA	–	Irregularity	Possible VBDA	Intimal flap/ double lumen	Irregularity	VBDA
6	Recurrent dizziness, 1460 days	Left ICVA	–	Aneurysmal dilation	Possible VBDA	Intimal flap/ double lumen/ IMH	Aneurysmal dilation	VBDA
7	Recurrent dizziness, 61 days	Left ICVA	–	Aneurysmal dilation with stenosis	Possible VBDA	Intimal flap/ double lumen/ IMH	Fusiform dilation	VBDA
		Right ICVA	–	Irregularity	Possible VBDA	Intimal flap/ double lumen	Irregularity	VBDA
8	Recurrent vertigo, 30 days	BA	–	Stenosis	Possible VBDA	Intimal flap/ double lumen	Fusiform dilation	VBDA
		Left ICVA	Intimal flap/double lumen	Aneurysmal dilation with stenosis	VBDA	IMH	Fusiform dilation with stenosis	VBDA
9	Recurrent headache, 365 days	Left ICVA	–	Aneurysmal dilation	Possible VBDA	Intimal flap/ double lumen/ IMH	Aneurysmal dilation	VBDA
10	Recurrent headache, 30 days	Left ICVA	–	Aneurysmal dilation	Possible VBDA	IMH	Aneurysmal dilation	VBDA
11	Recurrent dizziness, 60 days	BA	Intimal flap/double lumen	Fusiform dilation with stenosis	VBDA	Intimal flap/ double lumen	Fusiform dilation with stenosis	VBDA
12	Recurrent vertigo, 7 days	Left ICVA	–	Fusiform dilation with stenosis	Possible VBDA	Intimal flap/ double lumen/ IMH	Fusiform dilation with stenosis	VBDA
13	Coma, 4 h	Left ICVA	–	Aneurysmal dilation	Possible VBDA	Intimal flap/ double lumen	Aneurysmal dilation	VBDA
14	Dizziness and slurred vision, 30 days	BA	Intimal flap/double lumen	Stenosis	VBDA	Intimal flap/ double lumen	Irregularity	VBDA
		Left ICVA	–	Stenosis	Possible VBDA	Intimal flap/ double lumen/ IMH	Stenosis	VBDA
15	Recurrent headache, 1460 days	BA	–	Irregularity	Possible VBDA	Intimal flap/ IMH	Irregularity	VBDA
16	Dizziness, 1 day	Right ICVA	–	Aneurysmal dilation	Possible VBDA	Intimal flap/ IMH	Aneurysmal dilation	VBDA
17	Recurrent dizziness, 30 days	BA	Intimal flap/ double lumen	Aneurysmal dilation with stenosis	VBDA	Intimal flap/ double lumen/ IMH	Fusiform dilation with stenosis	VBDA
18	Dizziness, 60 days	BA	–	Fusiform dilation	Possible VBDA	Intimal flap/ double lumen	Fusiform dilation	VBDA
19	Recurrent headache, 365 days	Distal segment of right ICVA	Intimal flap/ double lumen	Fusiform dilation	VBDA	Intimal flap/ double lumen	Fusiform dilation	VBDA
		Proximal segment of right ICVA	Intimal flap/ double lumen	Aneurysmal dilation	VBDA	Intimal flap/ double lumen	Aneurysmal dilation	VBDA
		Left ICVA	–	Occlusion	Possible VBDA	IMH	Occlusion	VBDA
20	Limb numbness and weakness, 60 days	BA	–	Fusiform dilation	Possible VBDA	— (Normal wall thickness)	Fusiform dilation	Segmental ectasia
21	Dysphagia and right side weakness, 20 days	Right ICVA	–	Fusiform dilation	Possible VBDA	— (Normal wall thickness)	Fusiform dilation	Segmental ectasia

*VWI* vessel wall images, *BA* basilar artery, *IMH* intramural haematoma, *VBDA* vertebrobasilar dissecting aneurysm, *ICVA* intracranial vertebral artery

In contrast, based on VWI, all of the 27 lesions in the 21 patients were given a definite diagnosis, including 25 definite VBDAs in 19 patients, and 2 segmental ectasia in 2 patients (Fig. 2a-d). Thus, in this series, definitive diagnosis of a VDBA was more frequently made using VWI in comparison to catheter angiography (92.6%, 25/27 vs 25.9%, 7/27; *p* <  0.001) (Tables 1, 2). The definite diagnosis of segmental ectasia was only made by VWI, compared with catheter angiography (7.4%, 2/27 vs 0, 0/27). Furthermore, on the post-contrast VWI, all VBDAs showed extensive and dense wall enhancement. In contrast, segmental ectasias did not show obvious vessel wall enhancement.

### Direct signs of dissection

In this series of possible VBDAs, intimal flap sign was more frequently detected on VWI than on catheter angiography (77.8%, 21/27 vs 25.9%, 7/27; *p* = 0.001) (Figs. 1, 2g, k, o, 3d). Detection rate of double lumen sign was also higher on VWI (66.7%, 18/27 vs 25.9%, 7/27; *p* = 0.003) (Figs. 1, 2g, k, o, 3d). Furthermore, IMH sign was detected on VWI in 51.9% (14/27) of lesions, which is not detectable on catheter angiography (0/27). Of 14 lesions with IMH, 5 concomitantly had a partially thrombosed haematoma (Mizutani type 3 dissection, Fig. 2l, p).

### Comparison between early stage and chronic stage

On the VWI, there was no significant difference for the intimal flap sign, (76.5%, 13/17 vs 80.0%, 8/10; *p* = 1.000), double lumen sign (64.7%, 11/17 vs 70.0%, 7/10; *p* = 1.000), and the IMH (47.1%, 8/17 vs 60.0%, 6/10; *p* = 0.802) between the lesions in the early stage and chronic stage.

On the catheter angiography, there was also no significant difference for the intimal flap sign, (23.5%, 4/17 vs 30.0%, 3/10; *p* = 1.000), and double lumen sign (23.5%, 4/17 vs 30.0%, 3/10; *p* = 1.000) between the lesions in the early stage and chronic stage.

### Indirect signs of dissection

Based on the luminal imaging, there was no difference in the indirect signs of dissection between catheter angiography and HRMR VWI (*p* = 0.406). However, the HRMR VWI provided more detailed information including actual affected vessel length and dilated vessel surface appearance in all the 27 lesions.

## Discussion

Our study showed that HRMR VWI was superior to catheter angiography in detecting dissecting signs and achieving definite diagnosis of VBDA, and allowed a promising way to accurately discriminate between VBDA and segmental ectasia as well, by assessing vessel wall morphological and signal patterns in these various disease states.

Based on the pathological findings, Mizutani et al. classified nonatherosclerotic fusiform and dissecting aneurysms as type 1, classic dissecting aneurysm; type 2, segmental ectasia; type 3, dolichoectatic dissecting aneurysm; and type 4, saccular aneurysm at a non-branching site [10]. A better understanding of the pathological features of each lesion may help to establish individual treatment strategies, considering the strong relationship between the pathological patterns and their clinical courses [10]. Nowadays, given the difficulty in obtaining histopathological examination during clinical work, the superiority of HRMR VWI over catheter angiography for detecting pathognomonic radiological findings can help to differentiate between different types effectively.

An intimal flap has been regarded as the most reliable imaging finding for vascular dissection [3]. However, this can be a subtle sign, and only observed in a minority of cases with catheter angiography [2]. Our study showed that HRMR VWI with 3D SPACE sequence could detect intimal flaps in 21 of 27 lesions suspicious for VBDAs (77.8%), outperforming DSA, similar to results described in previous studies [3, 7]. For the other 4 VBDAs without detectable intimal flap on VWI, one reason may be due to healing of the dissection after the acute stage [1], resulting in intimal flap adherence to the parent vessel wall. A double lumen sign is also considered a reliable sign of vascular dissection. Detection of this sign relies on the identification of blood products in the pseudolumen [12, 13]. An entry-exit type dissection occasionally has a constant flow of blood through the pseudolumen, resulting in signal flow-void similar to the true lumen on MRI [12]. On the contrast, an entry-only type dissection often has an intramural haemotoma in the pseudolumen [12], leading to various signal intensities according to hemorrhagic age on MRI [8].

3D T1W images with high spatial resolution and black-blood effect is regarded as the optimum imaging method for detection of IMH [3]. The positive rate of IMH was 51.9% (14/27) in our patients. Intramural hematoma in VBDA was best detected during the subacute or early chronic stage; but after 2 months, IMHs are isointense and can become difficult to recognize on MR images at this age [8]. In some cases, IMH may be a recurrent process with both older and more acute blood products [9, 10], leading to the heterogenous, partially thrombosed haematoma, which can be found in fatal chronic dissecting aneurysm (Mizutani type 3) [9, 10]. This lesion is different from classic dissecting aneurysms (Mizutani type 1) regarding of the progress and outcomes, involving different therapeutic approach [13]. VWI could be valuable for discrimination between type 1 and 3 dissecting aneurysms, and directing therapeutic management.

Previous study [14] showed dissecting signs including intimal flap, double lumen sign, and IMH decreased significantly from the early stage to the chronic stage, which was different with our result. Some possible reasons could explain this. First, clinical symptoms in this study were not specific, and many patients showed recurrent TIA. Second, 5 patients had 2 or more dissecting aneurysms, and each aneurysm might happen in different stage in the same patient. Those reasons might confuse the result of the relationship between the stage and the image findings.

In this study, 2 cases presenting as local dilation without mural thrombosis, had wall thickness similar to the adjacent “normal” artery, and were diagnosed as segmental ectasias (Mizutani type 2). The natural history of this entity is still not well understood and somewhat controversial [10, 15]. Vessel wall enhancement on post-contrast VWI was widely detected on VBDAs, but not on segmental ectasias. Increased gadolinium enhancement might have important value for distinguishing definite dissections from possible ones. A larger sample and follow-up with VWI is required to assess the diagnostic value of vessel wall enhancement.

Multiple intracranial VBDAs were detected in 5 patients in this study. With 3D HRMR VWI, multiple lesions in the same patient with varying locations and orientations can be captured in one MR scan and reconstructed views can be obtained readily - which is difficult for the 2D VWI technique.

There are several limitations in our study. First, this study only included patients with suspicious of dissection on clinical symptoms and CTA or MRA, which may limit the generalizability of the results. Second, some new technique such as the delay alternating with nutation for tailored excitation (DANTE) could help improve blood suppression was not used in this study [16]. Residual flow artifacts might affect the assessment of intimal flap. Third, resolution (0.9 mm) may not be adequate for full assessment of VBDAs. Lastly and most importantly, the diagnoses could not be confirmed by histology, as all of the patients included in this study remain alive. Additionally, as the institution admits patients from a very large geographic area, many patients do not return for follow-up imaging or clinical evaluation limiting longitudinal analysis.

## Conclusions

3D HRMR VWI could detect direct dissection signs more frequently than catheter angiography. This may help obtain definite diagnosis of intracranial VBDAs, and allow accurate differentiation between dissecting aneurysm and segmental ectasia as well. Further prospective study with larger sample was required to investigate the superiority of HRMR VWI for definite diagnosis of intracranial VBDAs than catheter angiography.

## Data Availability

The datasets used and analysed during the current study are available from the corresponding author on reasonable request.

## Abbreviations

IMH: intramural haematomas
VBDAs: vertebrobasilar dissecting aneurysms
3D HRMR VWI: three-dimensional high-resolution magnetic resonance vessel wall imaging
2D FSE: two dimensional fast spin echo
SPACE: Sampling Perfection with Application optimized contrasts by using different flip angle Evolutions
CTA: computed tomographic angiography
IPH: Atherosclerotic intraplaque hemorrhage
TOF: time-of-flight
SAH: subarachnoid hemorrhage
DANTE: the delay alternating with nutation for tailored excitation
